# Fast and Efficient Mouse Pluripotency Reprogramming Using a Chemically-Defined Medium

**DOI:** 10.3390/mps5020028

**Published:** 2022-03-24

**Authors:** Junju Huang, Xuejie Yang, Jie Wang, Haoyu Wu, Duanqing Pei, Jiekai Chen

**Affiliations:** 1 Joint School of Life Sciences, Guangzhou Institutes of Biomedicine and Health, Chinese Academy of Sciences, Guangzhou Medical University, Guangzhou 511436, China; huang_junju@gibh.ac.cn; 2 CAS Key Laboratory of Regenerative Biology, Guangzhou Institutes of Biomedicine and Health, Chinese Academy of Sciences, Guangzhou 510530, China; yang_xuejie@gibh.ac.cn (X.Y.); wang_jie@gibh.ac.cn (J.W.); wu_haoyu@gibh.ac.cn (H.W.); 3 Guangdong Provincial Key Laboratory of Stem Cell and Regenerative Medicine, Guangzhou 510530, China; 4 Laboratory of Cell Fate Control, School of Life Sciences, Westlake University, Hangzhou 310024, China; 5 Center for Cell Lineage and Atlas (CCLA), Bioland Laboratory, Guangzhou Regenerative Medicine and Health GuangDong Laboratory, Guangzhou 510005, China

**Keywords:** reprogramming, induced pluripotent stem cells (iPSCs), transcription factors, mouse embryonic fibroblasts (MEFs), fast kinetics

## Abstract

The reprogramming of somatic cells to obtain induced pluripotent stem cells (iPSCs) is an important biological and medical breakthrough, providing important applications for fields such as regenerative medicine and disease modeling. However, this promising technology is damped due to its low efficiency and slow kinetics. Therefore, we generated a practical workflow to rapidly and efficiently induce iPSCs from mouse embryonic fibroblasts (MEFs) using iCD1 (iPS chemically-defined medium 1). This protocol can easily be implemented in a standard cell culture laboratory and be applied to cell fate research.

## 1. Introduction

A group of cells that are isolated from the inner cell mass at the blastocyst stage, termed pluripotent stem cells, are capable of in vitro self-renewal while maintaining their pluripotency. The blastocyst-derived pluripotent stem cells, named embryonic stem cells (ESCs), retain their differentiation potentials [1,2]. Their ability to differentiate into all adult cell types provides an avenue for developmental biology research and regenerative medicine [3].

Despite wide applications of ESCs, technical difficulties and ethical concerns impede the generation of human ESCs using the previous technologies such as somatic cell nuclear transfer [4] or cell fusion of somatic cells with the existing ESCs [5,6]. Since 2006, the ground-breaking discovery of mouse and human somatic reprogramming by overexpressing four transcription factors Oct4, Sox2, Klf4, and c-Myc (OSKM) [7,8,9,10,11] has substantially addressed the ethical difficulties.

The reprogramming of somatic cells into iPSCs with defined factors is a laborious and inefficient process. Usually, it takes two to three weeks to generate murine iPSCs from MEFs with the requirement of fetal bovine serum (FBS) and feeders to reach only ~0.05% efficiency [12]. Such an inefficient system greatly hinders molecular mechanism investigation of somatic reprogramming, and even the entry of laboratories in this field. Technically, the iPSC generation process includes cell preparation, factor delivery, cell fate induction, as well as culture of the reprogramming and reprogrammed cells. In our experience, a critical practice in cell preparation is the prevention of any mycoplasma contamination, an overlooked factor for the failure of reprogramming. The retroviral [7] and lentiviral [13] systems that are widely used in iPSC reprogramming are reliable and commercially available. We recommend the retroviral system as retrovirus transduction is much less cytotoxic than lentivirus. Therefore, the optimization of the reprogramming cell culture is a key process. By screening the chemicals and other components for cell culture, we developed a more efficient and fast way to generate iPSCs. We developed a feeder-independent culture medium termed iCD1 [14] that enhances the reprogramming efficiency up to 10% without the use of oncogene c-Myc.

Here we presented a stepwise method to generate iPSC and compared the efficiency of iCD1 with the traditional method using mES + Vitamin C induction medium. iPSCs can be generated within seven days. The iPSCs that are generated using this method are indistinguishable from embryonic stem cells in colony morphology, cellular functions, cell markers, and transcriptome and are thus suitable for downstream biological research [14]. Our method provides an easy-to-use system for mouse iPSC generation and serves as a starting point for further improvement of iPSC technology.

## 2. Experimental Design

**Figure 1:**
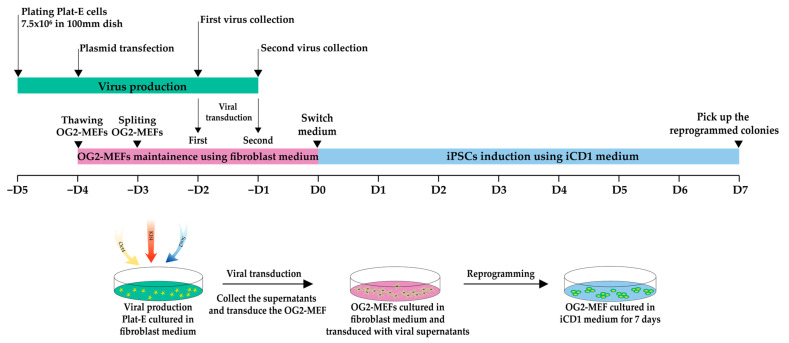
Workflow of iPSC generation from OG2-MEFs using iCD1. Timeline of viral production, OG2-MEFs preparation and transduction, and iPSC induction. Arrows indicate the key steps at different time points. OG2-MEFs (1 × 10^4^ cells) are split and plated in a 12-well plate.

Figure 1 shows the workflow of iPSC generation from OG2-MEFs using iCD1.

### 2.1. Materials

Table 1 provides all the consumables and equipment used in Section 3.

### 2.2. Reagents

Table 2 provides all the chemicals, antibodies, recombinant proteins, and plasmids used in Section 3.

### 2.3. Medium Recipes

Table 3 provides all the media, solution and their formula used in Section 3*;* Table 4 provides complete recipe of iCD1 use in Section 3.

### 2.4. Primers

Table 5 provides all the primers and their sequence used in real-time PCR.

### 2.5. Cell Lines

Table 6 provides all the cell lines used in Section 3.

## 3. Procedure

### 3.1. Viral Production through Calcium Phosphate Transfection

1. Plate 7.5 × 10^6^ or 4 × 10^6^ Plat-E cells to 100 mm or 60 mm cell culture dishes, respectively, depending on experimental design. The cells were cultured in fibroblast medium.
2. Observe the cells one day before performing calcium phosphate transfection and make sure the cells do not overgrow. The optimal confluence would be 70~80%.
3. Refresh with 7.5 mL and 2.5 mL media for 100 mm and 60 mm cell culture dishes 2 h before transfection, respectively.
4. Mix the following reagents in 1.5 mL or 15 mL tubes to prepare the cell transfection solution.

**Storage Con.**

**Usage Con.**

**60 mm Dishes**

**100 mm Dishes**
DNA1 μg/μL/8 μg25 μgH_2_O//429.5 μL1068.75 μLCaCl_2_2 M125 mM62.5 μL156.25 μL2× HBS2×1×500 μL1250 μLTotal//1000 μL2500 μL

Note: Add water first, then add DNA and mix thoroughly. Add 2 M CaCl_2_ and pipette 15 times. Finally, add 2× HBS and shake vigorously for 15 times to mix well (if the HBS is added multiple times, add it fast to avoid pH changes for the optimal transfection efficiency). Allow the mixed reagents to rest for 5 min at room temperature.

5. Uniformly add the mixed reagents to the cells and move back and forth and then side-to-side 1–3 times to ensure equal distribution. The calcium phosphate precipitation appears as black fine particles and can be observed throughout the dishes under a microscope. Place the cells back in a 37 °C incubator.
6. Replace the transfection medium with 10 mL or 3.5 mL fresh fibroblast medium for a 100 mm or 60 mm dish, respectively, 12 h after transfection.
7. Collect the supernatants that contain the viruses for the first transduction 48 h after transfection, and refresh with 10 mL or 3.5 mL fibroblast medium for a 100 mm or 60 mm dish, respectively.
8. Collect the supernatants with viruses 72 h after transfection for the second transduction.
9. Filter the virus-containing media with 0.45 μm filter and use the viruses freshly.

### 3.2. Thawing, Culturing, and Proliferating OG2-MEFs

1. Before thawing OG2-MEFs, coat the dish with 0.1% gelatin and incubate in a 37 °C incubator for at least 30 min.
  Note: OG2-MEFs should be tested for mycoplasma contamination as this will substantially impact the efficiency of iPSC generation.
2. Remove the vials from the liquid nitrogen using the appropriate safety equipment.
  Note: When handling frozen vials, make sure to wear appropriate personal protective equipment including cryo-gloves and eye protection as vials that are stored in liquid nitrogen may explode when warmed.
3. Immerse the vial in a 37 °C water bath without submerging the cap and keep swirling the vial gently.
  Note: The bottom of the vial that contains the OG2-MEFs should be immersed in the water bath; otherwise it would lead to cell death.
4. Remove the vial from the water bath when only a small piece of ice crystal is left as it will thaw within seconds due to the remaining heat.
5. Ensure that the cap is tight and spray the vial with 75% ethanol to sterilize the surface of the vial. Air-dry the vial in the sterile biosafety cabinet to ensure the absence of residual ethanol.
6. Transfer the cells into the bottom of a sterile 15 mL tube gently using a 1-mL pipette tip and add 5 mL fresh fibroblast medium dropwise with a 10 mL pipette.
  Note: If the medium is not added dropwise to make the cells adapt to the osmotic pressure of the medium, cell survival may be impacted due to the rapid change of the environment.
7. Centrifuge the cells at 200× *g* for 5 min.
8. Aspirate the supernatant and resuspend the cells with 1 mL fresh fibroblast medium gently.
9. Remove the 0.1% gelatin and slowly add 1 mL of the cell suspension into the 60 mm dish, followed by another 2 mL fresh fibroblast medium. Generally, 1 × 10^6^ OG2-MEFs are sufficient for three 60 mm dishes.
10. Feed 3 mL fibroblast medium to cells in one 60 mm dish every three days until ready to be passaged or harvested.

### 3.3. Passaging, Counting, and Plating OG2-MEFs

1. OG2-MEFs are split when they reach 80~90% confluence. Wash the cells with 3 mL DPBS, as the serum in the fibroblast medium contains a massive amount of trypsin inhibitors.
2. Aspirate DPBS, then add 0.5 mL pre-warmed 0.25% trypsin-EDTA, and incubate at 37 °C for 2 min.
  Note: The best working temperature for trypsin is 37 °C. Pre-warmed trypsin can fully disassociate OG2-MEFs in 2 min. Long and short incubation will lead to poor cell vitality and residual cells, respectively.
3. When OG2-MEFs are well detached as single cells, add an equal volume of fibroblast medium to terminate the trypsin reaction.
4. Transfer the cell suspension to a sterile 15 mL tube. Take 10 µL of the cell suspension for cell counting.
5. Centrifuge the cells at 200× *g* for 5 min at room temperature. Meanwhile, count the cells using a hemocytometer or an automated cell counter.
  Note: Cell concentrations between 4–6 × 10^5^ would give a more accurate result when using a hemocytometer.
6. Aspirate the supernatants and resuspend the cells first with 1 mL medium. Pipette up and down to ensure a single-cell suspension. Then add a proper medium volume to obtain 1.5 × 10^4^ living cells per well in 12-well plates.
  Note: Gently shake the tube to ensure equal distribution of the cells and an equal number of cells in every well. Normally, 1.5–2 × 10^4^ cells are recommended for OKS reprogramming and 3 × 10^4^ cells are recommended for OK/OS/O reprogramming.
7. Gently move the plates back-and-forth to ensure uniform distribution of the cells before placing them back in the incubator.
  Note: Due to the edge effect of the plate, rotational movement should be avoided to ensure that cells do not aggregate in the middle of the well. Close the incubator gently to avoid disturbance.
8. OG2-MEFs will completely adhere to the well in 8 h which is the appropriate window for viral transduction, as poor adhesion of OG2-MEFs will lower the reprogramming efficiency.

### 3.4. Generation of iPSCs

1. Aspirate the spent medium and add 0.5 mL of each virus-containing media, which were collected and filtered from Section 3.1 followed by adding 0.5 mL fibroblast medium containing 4 µg/mL polybrene. Normally, the volume of the virus-containing media depends on the transfection efficiency in Plat-E cells during viral production in Section 3.1. It is recommended to use 0.5 mL virus-containing media if the efficiency achieves 90%, and to use 0.75 mL virus-containing media if the efficiency is 80% or lower.
2. Perform the second transduction by repeating step 1 one day after the primary transduction.
3. The virus-containing media are removed 24 h after the second transduction and 1 mL of iCD1 is added to the cells. The day on which virus-containing media are removed is denoted as day 0 post-transduction.
4. Feed the cells daily with 1 mL fresh medium per well of a 12-well plate, 2 mL at day 5 and hereafter, as the robust proliferation of cells will lead to a deficiency of nutrition during reprogramming.
  Note: Medium should be pre-warmed at room temperature in the dark before use, as the small molecules in the medium are photolytic. Use the medium freshly (within a week) as it contains reducing substances and many unstable growth factors.
5. Oct4-GFP positive and dome-shaped colonies are considered as reprogrammed iPSC colonies.
6. The iPSC colonies are picked at day 7 post-transduction based on Oct4-GFP expression and characteristic ESC-like morphology. The picked colonies are subsequently expanded and maintained the same way as ESCs.

### 3.5. Characterization of iPSCs by Immunostaining (in a Cell Culture Plate)

1. Gently remove the medium and wash the cells with 1 mL of DPBS twice.
2. Add 1 mL of 4% (wt/vol) PFA to each well of a 12-well plate and let it stand at room temperature for 30 min. Gently replace with 1 mL of DPBS and shake for 5 min at room temperature. Repeat the DPBS wash twice.
3. Add 500 μL blocking and permeabilization solution (one volume of 3% GSA and one volume of 0.2% triton mixed together). Incubate for 40 min at 25 °C.
4. Wash the well with 1 mL of DPBS, place it on a horizontal shaker at room temperature and shake for 5 min. Repeat the DPBS wash twice.
5. Incubate with the Anti-Nanog antibody (1:1000) at 4 °C overnight.
6. Wash the cells with DPBS thrice and incubate for 10 min each time at room temperature on a horizontal shaker.
7. Incubate with the secondary antibody (1:2000) for 1 h at room temperature and avoid light.
8. Wash the cells with DPBS thrice and incubate for 10 min each time at room temperature on a horizontal shaker (avoid light).
9. Add 1 mL DPBS into each well and use a fluorescence microscope to capture images.

## 4. Expected Results

**Figure 2:**
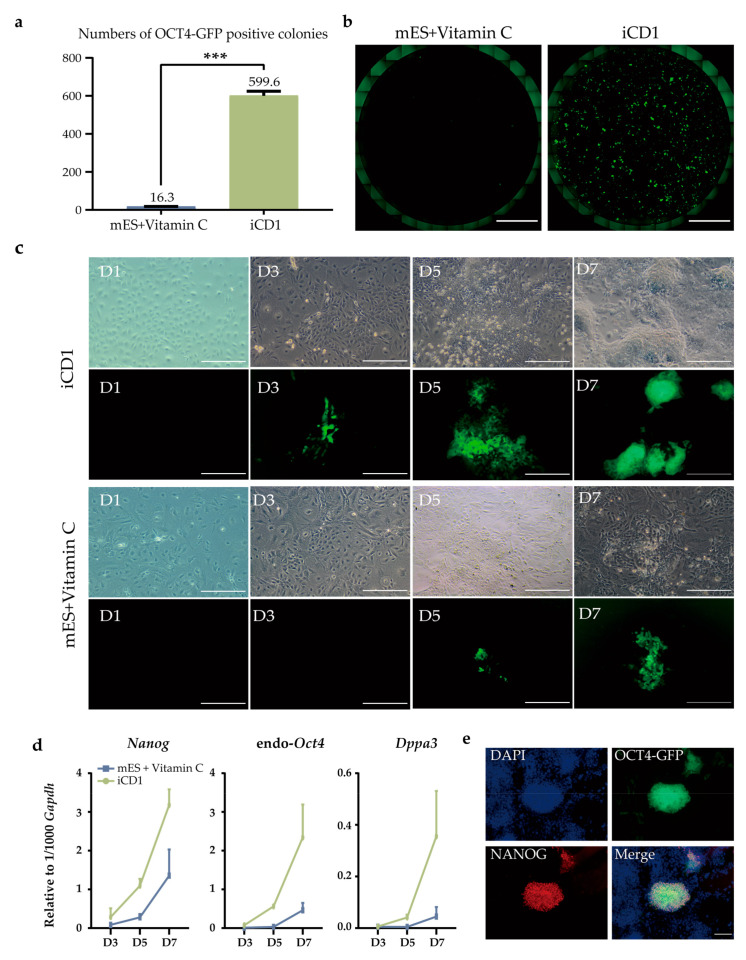
Characterization of generated iPSCs using the iCD1 medium. (**a**) Oct4-GFP-positive colonies were scored at day 7 post-treatment in indicated medium. *** *p* < 0.001, compared to the result of iCD1 (n = 2); (**b**) Images of Oct4-GFP positive colonies generated by indicated medium at day 7. Scale bar: 5mm; (**c**) The induction of pluripotency from MEFs by *Oct4* (O), *Klf4* (K), *Sox2* (S) in the indicated medium during reprogramming. The bright field and GFP images were shown on the top and bottom, respectively. Green colonies indicate successful reprogramming. Scale bar: 250 μm; (**d**) Expression levels of *Nanog*, endogenous *Oct4* and *Dppa3* were analyzed by quantitative real-time PCR (n = 2). Expression levels of pluripotent markers were relative to *Gapdh*; (**e**) Immunofluorescent staining of NANOG in Oct4-GFP positive iPSCs, showing a co-expression of OCT4 and NANOG proteins in GFP positive cells. Scale bar: 250 μm.

We treated OG2-MEFs with the modified medium iCD1 and traditional induction medium mES + Vitamin C and found that the number of Oct4-GFP-positive colonies was substantially increased in iCD1. The number of Oct4-GFP-positive colonies at day seven post-treatment was almost 40-fold higher than that in mES + Vitamin C medium (Figure 2a,b). We observed that mesenchymal-epithelial transition (MET) occurred the day after the second transduction (D1). The iCD1-treated cells activated Oct4 sooner than the mES + Vitamin C-treated ones and as reprogramming progressed. The iCD1-treated cells exhibited round shape, large nucleoli, and sparse cytoplasm similar to mouse ESCs (Figure 2c). We collected RNA samples of the cells that were treated by both conditions at day three, five, and seven, followed by quantitative real-time PCR to investigate the reprogramming kinetics using primers as stated in Table 4. We found that the iCD1-treated cells exhibited earlier activation of pluripotent markers, *Nanog*, endogenous *Oct4*, and *Dppa3,* than those by mES + Vitamin C, indicating the advantage of iCD1 in reprogramming kinetics (Figure 2d). Next, we examined the NANOG expression of iCD1-treated cells by immunostaining using NANOG antibody. The Oct4-GFP-positive cells were also found to be NANOG-positive, further confirming the pluripotency establishment (Figure 2e). Additional characterization of iCD1-induced iPSC can be found in our previous works [14]. Altogether, the upregulation of pluripotent markers indicates a successful cell fate transition and well-established pluripotency of the reprogrammed cells.

## 5. Discussion

The technical improvement of iPSC reprogramming has mainly focused on reducing or replacing transcription factors (e.g., subtracting the oncogene c-Myc) [15,16]. Yet this strategy suffers from low efficiency and lengthy process compared to the iCD1 system that is described here. Alternatively, non-integrated delivery methods of reprogramming factors such as mRNA delivery and transient transfection make iPSCs more clinically applicable [17,18]. Subsequent studies have reported uses of many mouse cell types for reprogramming such as neural progenitor cells, pancreatic β cells, and stomach cells, thereby expanding the choices of the starting cell types [19,20,21,22]. Presently, more efforts are focused on adding small molecular compounds to replace transcription factors. For instance, Huangfu et al. reported that histone deacetylase (HDAC) inhibitors, particularly valproic acid (VPA), markedly improve the reprogramming efficiency [23]. Vitamin C is a dioxygenase agonist, and its remarkable role in promoting reprogramming efficiency is well documented [24]. It is noted that protocols using Knockout serum replacement (KSR)-based medium should be considered as Vitamin C-supplemented protocols, as KSR enriches Vitamin C. Hou et al. reported a successful induction of iPSC by replacing all the transcription factors with compound combinations [25]. As chemical-induced reprogramming is inefficient and requires a tedious process, optimized protocols have been developed for high efficiency and reproducibility [26,27].

However, these methods have their shortcomings. Transient transfection has delayed kinetics of reprogramming and short expression window of factors compared to the integrated expression [18]. Many reported chemicals such as 5′-azaC and BIX01294 are cytotoxic and not conducive to application [28,29]. Moreover, small molecules that replace transcription factors may have very low efficiency, a long induction time, and undefined side effects or toxicity on cells [23].

We have developed a user-friendly protocol allowing for fast and efficient reprogramming of MEFs into iPSCs in iCD1, an original reprogramming medium. Our lab has previously developed a serum-free medium containing multiple vitamins and engineered factors that improve reprogramming efficiency after testing candidate factors systematically [29,30]. The previously reported complex formulation can be substituted by iCD1, containing high glucose DMEM-supplemented N2, B27, and vitamin C. Moreover, the addition of small molecules GSK-3β inhibitor CHIR99021 and LiCl into our iCD1 medium maintains the undifferentiated state of already reprogrammed iPSCs while accelerating somatic cell reprogramming [31]. The growth factor, basic fibroblast growth factor (bFGF), is crucial to support OKS-transduced MEFs proliferation and later-stage reprogramming. Further, we used MEFs that were derived from E13.5 embryos carrying the Oct4-GFP transgenic allele [32], easy for tracing the kinetics of the reprogramming. We applied a retroviral delivery system as it is susceptible to transgene-silencing at the fully reprogrammed iPSC stage. We found that iCD1 supplemented with BMP factors can support Oct4-mediated or Oct4-Sox2-mediated reprogramming by promoting MET process, the lack of which is a major hindrance in reprogramming without Klf4 and c-Myc [33].

In general, iCD1 has extensive application in somatic reprogramming and has already been used and modified in many studies [34,35,36] to further decipher cell fate decision.

## 6. Conclusions

Here, we provide a detailed procedure of efficient and fast mouse iPSC generation using a chemically-defined medium that was developed in our lab previously. The resulting iPSC colonies have morphological and molecular signatures as ES cells. Our protocol has provided a reliable and efficient way to comprehensively study molecular mechanisms of cell fate transition and facilitate the application of reprogramming technologies.

## Figures and Tables

**Table 1:** Information on all consumables and equipment.

Name	Source	Identifier	Location
Pipette tips 10 μL	Corning	T-300	Glendale, AZ, USA
Pipette tips 200 μL	Corning	T-200-Y	Glendale, AZ, USA
Pipette tips 1 mL	Corning	T-1000-B	Glendale, AZ, USA
Microtubes	Corning	MCT-150-C	Glendale, AZ, USA
15 mL tubes	Corning	430790	Glendale, AZ, USA
50 mL tubes	Corning	430828	Glendale, AZ, USA
Cell culture multiwell plate, 6 well	Greiner	657160	Kremsmünster, Austria
Cell culture multiwell plate, 12 well	Greiner	665180	Kremsmünster, Austria
60 mm cell culture dishes	Greiner	664160	Kremsmünster, Austria
100 mm cell culture dishes	Greiner	628160	Kremsmünster, Austria
0.45 μm filter	Millipore	SLHVR33RB	Burlington, MA, USA
4 °C refrigerator	Haier	HYCD-290	Guangzhou, China
−20 °C freezer	Haier	HYCD-290	Guangzhou, China
−80 °C freezer	Thermo Fisher Scientific	995	Waltham, MA, USA
Heracell 240i Incubator	Thermo Fisher Scientific	51026331	Waltham, MA, USA
Microscope	ZEISS	Vert.A1	Oberkochen, Germany
Low-speed centrifuge	ZONKIA	SC-3612	Anhui, China
QuantStudio^TM^ 3 Real-Time PCR Instrument	Applied Biosystems	A28132	Waltham, MA, USA

**Table 2:** Information on all chemicals, antibodies, recombinant proteins, and plasmids.

Name	Source	Identifier	Location
Dulbecco’s Phosphate-Buffer Saline (DPBS)	HyClone	SH30028.02	Logan, UT, USA
DMEM High Glucose	Hyclone	SH30022.01	Logan, UT, USA
FBS (for mES + Vitamin C medium)	Lonsera	S711-001s	Shanghai, China
FBS (for fibroblast medium)	NATOCOR	SFBE	Córdoba, Argentina
GlutaMAX	GIBCO	35050079	Waltham, MA, USA
Non-Essential Amino Acids Solution (NEAA)	GIBCO	11140076	Waltham, MA, USA
Sodium Pyruvate	GIBCO	11360070	Waltham, MA, USA
β-Mercaptoethanol	GIBCO	21985-023	Waltham, MA, USA
Trypsin-EDTA (0.25%)	GIBCO	25200114	Waltham, MA, USA
DAPI	Sigma	D9542	Burlington, MA, USA
GSA	ZSGB-BIO	ZLI-9022	Beijing, China
Triton x-100	Sigma	T9284	Burlington, MA, USA
Nanog Polyclonal Antibody	BETHYL	A300-397	Montgomery, TX, USA
Alexa Fluor 568 Goat anti-Rabbit	Invitrogen	A11011	Waltham, MA, USA
ChamQ^TM^ SYBR qPCR Master Mix kit	Vazyme	Q311	Nanjing, China
HiScript II Q RT SuperMix for qPCR kit	Vazyme	R222-01	Nanjing, China
TRI Reagent	MRC	TR118-200	Cincinnati, OH, USA
CHIR99021	Sigma	SML1046	Burlington, MA, USA
Thiamine hydrochloride	Sigma	T1270	Burlington, MA, USA
2-Phospho-L-ascorbic acid trisodium salt (Vitamin C)	Sigma	49752	Burlington, MA, USA
Lithium chloride	Sigma	L4408	Burlington, MA, USA
Polybrene	Sigma	TR1003	Burlington, MA, USA
Sodium phosphate dibasic	Sigma	S7907	Burlington, MA, USA
Potassium chloride	Sigma	P9333	Burlington, MA, USA
HEPES	Sigma	H7523	Burlington, MA, USA
D-(+)-Glucose	Sigma	G6152	Burlington, MA, USA
Sodium chloride	Sigma	S5886	Burlington, MA, USA
Mouse leukemia inhibitory factor (LIF)	Millipore	ESGE107	Burlington, MA, USA
pMX-Oct4	Addgene	13366	Watertown, MA, USA
pMX-Sox2	Addgene	13367	Watertown, MA, USA
pMX-Klf4	Addgene	13370	Watertown, MA, USA
pMX-DsRed	Laboratory of D. Pei	N/A	Guangzhou, China

**Table 3:** Detailed information on the media and solution formula.

Name	Recipe
2× HBS (500 mL)	NaCl 8.1816 g, KCl 0.8715 g, Na_2_HPO4 0.10647 g, Glucose 1.08096 g, HEPES 5.95775 g, adjust PH to 6.92–6.95 with NaOH, add ultrapure water to 500 mL
2 M CaCl_2_ (500 mL)	CaCl_2_ 147.02 g, add ultrapure water to 500 mL
Fibroblast medium	DMEM/high glucose 500 mL, FBS (NATOCOR) 10% (56 mL), NEAA 1/100 (5.6 mL), GlutaMAX 1/100 (5.6 mL)
mES + Vitamin C	Lonsera FBS 7.5 mL, NEAA 500 μL, GlutaMAX 500 μL, Sodium Pyruvate 500 μL, β-Mercaptoethanol (55 mM) 91 μL (Final concentration 0.1 μM), 2-Phospho-L-ascorbic acid trisodium salt (Vitamin C, final concentration 50 μg/mL) 50 μL, Mouse leukemia inhibitory factor (0.1 mg/mL, Final concentration 12.5 ng/mL) 6.25 μL, add DMEM/high glucose to make the volume 50 mL
iCD1	The recipe is shown in Table 4
3% GSA Blocking Buffer	GSA 1.5 mL, DPBS 48.5 mL
0.2% Permeabilization Buffer	Triton 0.1 mL, DPBS 49.9 mL

**Table 4:** Components of the iCD1 medium.

Substance	mg/L	Substance	mg/L
L-Arginine·HCl	8.40 × 10^1^	Arachidonic adic	2.00 × 10^−2^
L-Alanine	8.90	Cholesterol	2.20
L-Asparagine	1.32 × 10^1^	Linoleic acid	1.00 × 10^−1^
L-Aspartic acid	1.33 × 10^1^	Linolenic acid	1.00 × 10^−1^
L-Cystine·2HCl	6.30 × 10^1^	Myristic acid	1.00 × 10^−1^
L-Glutamic acid	1.47 × 10^1^	Oleic acid	1.00 × 10^−1^
L-Histidine HCl·H20	4.20 × 10^1^	Palmitoleic acid	1.00 × 10^−1^
L-Isoleucine	1.05 × 10^2^	Palmitic acid	1.00 × 10^−1^
L-Leucine	1.05 × 10^2^	Pluronic F-18	1.00 × 10^3^
L-Lystine HCl	1.46 × 10^2^	Stearic acid	1.00 × 10^−1^
L-Methionine	3.00 × 10^1^	Tween 80	2.20 × 10^1^
L-Phenylalanine	6.60 × 10^1^	2-Phospho-L-ascorbic acid	5.00 × 10^1^
L-Proline	1.15 × 10^1^	D,L-alpha-tocopherol(Vitamin E)	1.00
L-Serine	6.30 × 10^1^	D,L-alpha-tocopherol acetatec	1.00
L-Threonine	9.50 × 10^1^	Biotin	1.00 × 10^−1^
L-Tryptophan	1.60 × 10^1^	D-Ca pantothenate	4.00
L-Tyrosine·2Na·2H2O	1.04 × 10^2^	Choline chloride	4.00
L-Valine	9.40 × 10^1^	Folic acid	4.00
Glycine	4.50 × 10^1^	i-Inositol	7.20
D-Glucose	4.50 × 10^3^	Niacinamide	4.00
Sodium pyruvate	1.10 × 10^2^	Pyridoxine HCl	4.00
D(+)-Galactose	1.50 × 10^1^	Riboflavin	4.00 × 10^−1^
Insulin(Bovine, Recombinant)	5.00 × 10^1^	Thiamine HCl	4.00
Transferrin(Human)	1.00 × 10^2^	Retinol, all trans(Vitamin A)	1.00 × 10^−1^
Recombinant BSA Fraction V	1.00 × 10^3^	Vitamin B12	1.40
Catalase	2.50	Putrescine·2HCl	3.22 × 10^1^
Glutathione(Reduced)	1.50	L-Carnitine HCl	2.00
Superoxide dismutase	2.50	Ethanolamine HCl	1.00
T-3/Albumin Complex	2.00 × 10^−3^	Lipoic acid	4.70 × 10^−2^
Corticosterone	2.00 × 10^−2^	Phenol red	1.50 × 10^1^
Progesterone	1.26 × 10^−2^	CHIR99021	1.40
basic FGF	5.00 × 10^−3^	2-mercaptoethanol	8.17
Leukemia inhibitory factor	1.00 × 10^−2^		
LiCl	2.12 × 10^2^		
CaCl_2_(anhyd.)	2.00 × 10^2^		
Fe(NO_3_)_3_·9H_2_0	1.00 × 10^−1^		
KCl	4.00 × 10^2^		
MgSO_4_	9.77 × 10^1^		
NaCl	6.40 × 10^3^		
NaHCO_3_	3.70 × 10^3^		
NaH_2_PO_4_·H_2_O	1.25 × 10^2^		
Sodium selenite	1.86 × 10^−2^		

**Table 5:** Information on the primers.

Name	Sequence (5′ to 3′)
*Gapdh-*F	AACTTTGGCATTGTGGAAGGGCTCA
*Gapdh-*R	TTGGCAGCACCAGTGGATGCAGGGA
*Nanog-*F	CTCAAGTCCTGAGGCTGACA
*Nanog-*R	TGAAACCTGTCCTTGAGTGC
Endo*-Oct4-*F	TAGGTGAGCCGTCTTTCCAC
Endo*-Oct4-*R	GCTTAGCCAGGTTCGAGGAT
*Dppa3-*F	TGTGGAGAACAAGAGTGA
*Dppa3-*R	CTCAATCCGAACAAGTCTT

**Table 6:** Detailed information on the cell lines.

Name	Source
Platinum-E (Plat-E)	A gift from The Fourth Military Medical University
OG2 Mouse Embryonic Fibroblast	E13.5 mouse embryos from crossing male Oct4–GFP transgenic mice (CBA/CaJ XC57BL/6J) to 129Sv/Jae female mice

## Data Availability

The data that are presented in this study are available within the figures.
